# Effects of Basil Seed Gum on Physicochemical, Antioxidant, and Sensory Properties of Carrot Powder‐Enriched Pancakes

**DOI:** 10.1002/fsn3.72255

**Published:** 2026-08-12

**Authors:** Fakhreddin Salehi, Sepideh Vejdanivahid

**Affiliations:** ^1^ Department of Food Science and Technology, Faculty of Food Industry Bu‐Ali Sina University Hamedan Iran

**Keywords:** basil seed gum, carrot pancakes, hardness, moisture, total phenolic content

## Abstract

Natural hydrocolloids are increasingly used in bakery products to improve texture, stability, and functional quality. This study evaluated the effect of basil seed gum (BSG) at levels of 0, 0.1%, 0.2%, and 0.3% (w/w) on the quality characteristics of carrot powder‐enriched pancakes. The methodology involved a comprehensive analysis of various parameters, including color attributes, weight, volume, density, texture (puncture test), moisture content, ash content, pH, acidity, total phenolic content (TPC), antioxidant capacity (AC), and sensorial evaluation, utilizing established analytical techniques and digital image processing. BSG incorporation significantly increased batter lightness and intensified redness and yellowness (*p* < 0.05), while in baked pancakes it caused a slight darkening of the crust and simultaneously enhanced core lightness and color saturation. Baking loss decreased significantly from 8.32% in the control to 1.48% at 0.3% BSG, while pancake volume increased from 23.02 to 26.58 cm^3^ and density decreased from 995.54 to 926.87 kg/m^3^ (*p* < 0.05). Hardness was reduced from 0.56 N in the control to 0.35 N at the highest BSG level, accompanied by a substantial increase in moisture content from 36.49% to 47.58%. The pH decreased from 6.97 to 6.40, whereas acidity increased from 0.20% to 0.46% with increasing BSG concentration. TPC increased from 983.88 to 1019.65 μg GAE/g, and AC improved from 74.06% to 75.73%. Sensory evaluation showed that texture, flavor, and overall acceptability scores increased with BSG addition, reaching their highest values at 0.3%, despite a reduction in appearance scores. In conclusion, BSG effectively enhanced the physical quality, sensory acceptance, and functional properties of carrot pancakes and can be considered a promising natural hydrocolloid for bakery applications.

## Graphical abstract

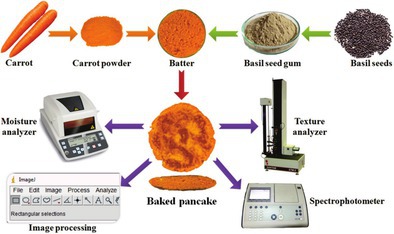

## 1 Introduction

Bakery products are a significant component of the human diet, providing essential energy, nutrients, and sensory satisfaction (Sarmasti et al. 2023). Among these, pancakes are widely consumed due to their convenience, palatability, and versatility in incorporating functional ingredients (Ozcelik et al. 2024; Salehi and Vejdanivahid 2025a; Samary et al. 2025b). However, the incorporation of plant‐based powders, such as carrot powder, into pancake formulations often poses challenges related to texture, moisture retention, color, and overall consumer acceptability. Carrot ( *Daucus carota* L.) is rich in carotenoids, dietary fiber, vitamins, and bioactive compounds with antioxidant properties, making it an attractive functional ingredient for the development of nutritionally enhanced bakery products (Kelki Bakhshayesh et al. 2019; Kırbaş et al. 2019; Salehi and Aghajanzadeh 2020; Jamal Asl et al. 2024; Kausar et al. 2024). Nevertheless, the addition of carrot powder can alter the structural and sensory characteristics of pancakes, leading to decreased volume, increased hardness, and heterogeneous core structure, which may limit consumer acceptance (Kırbaş et al. 2019; Salehi and Aghajanzadeh 2020).

Hydrocolloids have been extensively employed in bakery formulations to overcome such challenges (Dehghani Firoozabadi et al. 2012; Salehi 2020). These polysaccharide‐based biopolymers can improve water retention, gas‐holding capacity, and overall dough or batter rheology, thereby enhancing the texture, volume, and shelf‐life of baked products (Chen et al. 2022). Dehghani Firoozabadi et al. (2012) evaluated the effects of psyllium mucilage at concentrations of 0.05%, 0.1%, and 0.15% on the sensory properties of sponge cakes. They reported that the incorporation of psyllium gum enhanced sensory attributes and improved the physical characteristics of the final product.

Among natural hydrocolloids, basil seed gum (BSG), derived from *Ocimum basilicum* L., has emerged as a promising ingredient due to its high water‐holding capacity, gel‐forming ability, and functional bioactive compounds (Guan et al. 2023; Shahrajabian and Sun 2023). BSG contains polysaccharides, proteins, and phenolic compounds, which not only improve technological properties but also contribute to the nutritional and antioxidant profile of food products (Peighambardoust et al. 2016; Salehi 2020). BSG has been extensively investigated and applied in various fields, including food, pharmaceutical, and industrial applications, due to its functional and physiological properties. It is recognized as a valuable source of dietary fiber with potential health benefits, such as supporting weight management and contributing to the regulation of blood glucose and cholesterol levels (Guan et al. 2023). Previous studies have demonstrated the potential of BSG in improving the texture, moisture retention, and bioactive content of bakery and confectionery items (Asgari Verjani et al. 2021; Ghaemi et al. 2022; Salehi and Vejdanivahid 2025a); however, its application in carrot‐enriched pancakes has not been comprehensively investigated.

Color and sensory attributes are crucial determinants of consumer acceptance for bakery products. The incorporation of hydrocolloids can influence batter and baked product color, crust formation, and core lightness, which, in turn, affect hedonic perception (Salehi and Vejdanivahid 2025a). Additionally, functional fortification with hydrocolloids may enhance bioactive compounds, such as total phenolic content (TPC) and antioxidant capacity (AC), thereby offering health‐promoting benefits beyond basic nutrition (Asgari Verjani et al. 2021; Shahrajabian and Sun 2023). Despite the growing interest in clean‐label functional ingredients, there remains limited systematic research addressing the combined impact of BSG on the physicochemical, bioactive, and sensory characteristics of carrot‐enriched pancakes.

Therefore, the present study aimed to evaluate the effects of different concentrations of BSG (0%, 0.1%, 0.2%, and 0.3%, w/w) on the quality attributes of carrot powder–enriched pancakes. Specifically, the study investigated: (i) changes in color parameters of batter and baked pancakes, (ii) physical properties including weight, baking loss, volume, density, and hardness, (iii) chemical characteristics such as moisture content, pH, titratable acidity, and ash content, (iv) bioactive properties including TPC and AC, and (v) sensory acceptance. The findings are expected to provide valuable insights into the functional application of BSG as a natural hydrocolloid for improving both the technological performance and nutritional quality of pancake products.

## 2 Materials and Methods

### 2.1 Preparation of Carrot Powder

The carrot samples used in this study were collected in compliance with national regulations and with official authorization from the Iranian National Standards Organization. Fresh carrots, sourced from a cultivation site in Hamedan, Iran, were meticulously cleaned and subsequently sliced into uniform 1 mm thick sections utilizing a manual grating apparatus. The sliced samples were dehydrated in a laboratory oven (K.M 55, Pars Azma Co., Isfahan, Iran) at 70°C for 70 min. After drying, the carrots were milled using a high‐power industrial grinder (Best, 1600 W, China). The resulting powder was finally passed through a 60‐mesh sieve to obtain a uniform particle size distribution.

### 2.2 Materials

Basil seeds ( *Ocimum basilicum* L.) were procured from a local market in Hamedan, Iran. Following procurement, the seeds were thoroughly cleaned. Subsequently, they were soaked in distilled water (pH 7.0) at a water‐to‐seed ratio of 20:1 and a temperature of 25°C for 20 min to facilitate hydrocolloid release. The mucilage layer was then separated from the swollen seeds using a specifically designed extractor featuring a rotating plate mechanism to scrape the gum layer from the seed surface. The resulting BSG dispersions were dried in a forced‐air oven at 70°C for 48 h. The dried BSG was then milled and sieved through a 35‐mesh sieve (0.50 mm diameter). Finally, the milled powder was weighed and stored in airtight containers for subsequent analysis (Salehi and Inanloodoghouz 2023).

Vanilla (Hamishak, Iran), baking powder (Miyofel, Iran), sugar (Zamen, Iran), pasteurized whole milk (3.4% fat; Pegah, Iran), fresh eggs (Telavang, Iran), and cooking oil (Tabiat, Iran) used in the formulation of carrot pancakes were purchased from retail stores in Hamedan, Iran.

### 2.3 Pancake Preparation

Following an extensive review of previous studies and preliminary experimental evaluations (Salehi and Vejdanivahid 2025a, 2025b), an optimized formulation for carrot pancakes was established. The formulation consisted of 70 g carrot powder, 1 g vanilla, 4 g baking powder, 25 g sugar, 100 g pasteurized whole milk, 57 g fresh egg, and 6 g oil. Initially, egg whites were whipped for 2 min using an electric mixer until a stable foam was formed. In a separate bowl, egg yolks and vegetable oil were mixed for 1 min to obtain a homogeneous emulsion. Subsequently, 50 g of milk was added and mixed again. The dry ingredients (including carrot powder, sugar, vanilla, BSG, and baking powder), which had been previously sieved, were then incorporated into the mixture and mixed for 2 min. The remaining 50 g of milk was added, and mixing was continued for an additional 3 min. Next, the whipped egg white foam was gently folded into the batter and mixed for 1 min. The prepared batter was allowed to rest at ambient temperature for 10 min. For baking, a pan preheated to 180°C–190°C was used, and 25 g portions of batter were cooked for 5 min on one side and 2 min on the other side. Finally, the baked pancakes were cooled to room temperature and packaged in polyethylene containers with low oxygen and moisture permeability.

**FIGURE 1:**
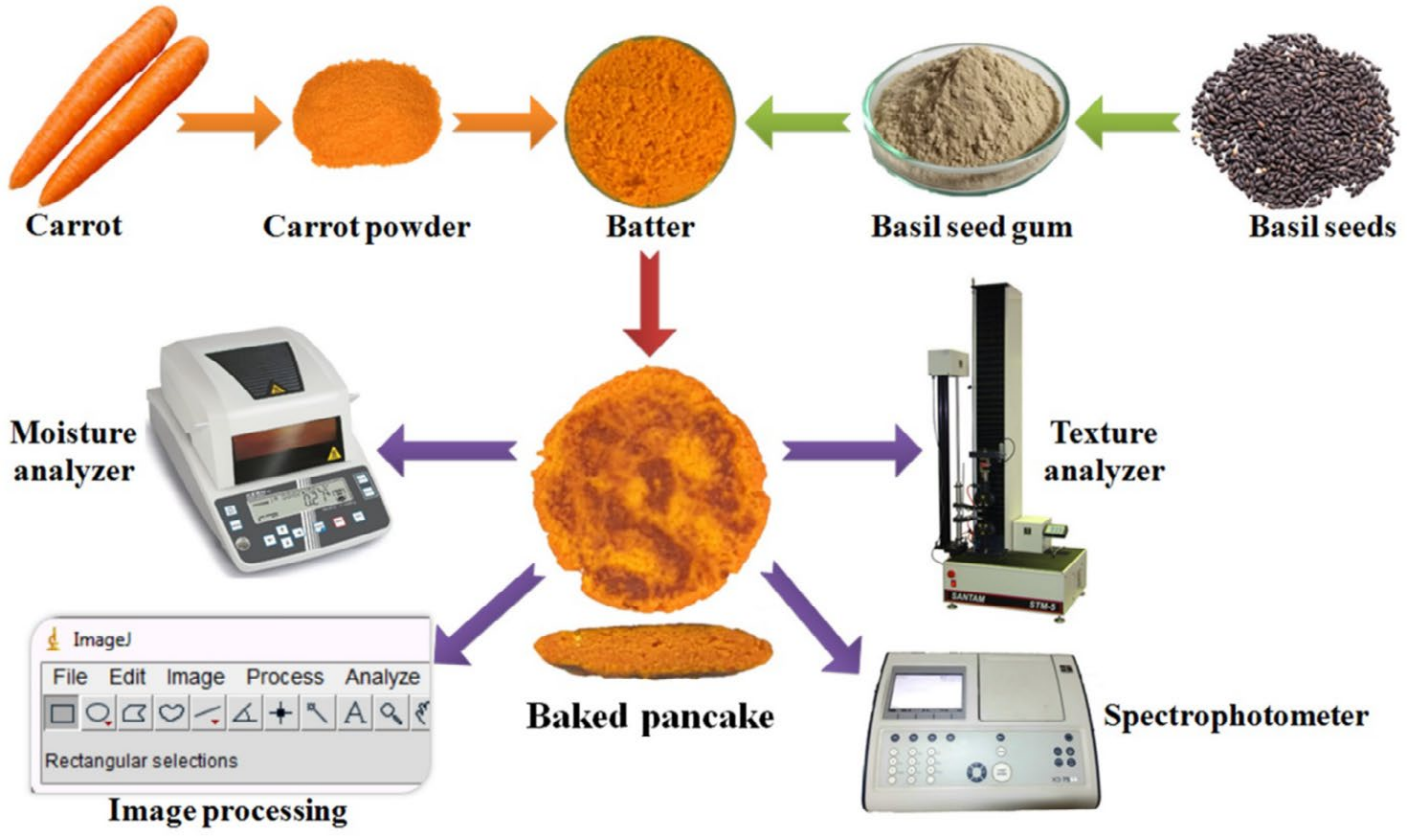
Graphical overview of pancake formulation incorporating carrot powder and basil seed gum and evaluation of physicochemical properties.

BSG was incorporated into the pancake formulation at four levels (0%, 0.1%, 0.2%, and 0.3%, w/w) based on the total formulation (Figure 1). To maintain a constant overall formulation and avoid changes in ingredient proportions, the amount of BSG added was compensated by an equivalent reduction in the vegetable oil content.

### 2.4 Color Parameters Determination

Color attributes of the pancake batter as well as the outer surface (crust) and core (crumb or internal structure) of the pancakes were assessed using image analysis methods. Sample images were captured with a 48‐megapixel digital camera (iPhone 15 Pro Max, Apple Co., China). The recorded images were subsequently processed in ImageJ software (version 1.42e, USA), where the RGB color data were transformed into L*, a*, and b* color coordinates using a dedicated plugin (Samary et al. 2025b).

### 2.5 Weight and Baking Loss Determination

The mass of the baked carrot pancakes was determined using a digital scale (GM‐300p, Lutron, Taiwan) with an accuracy of 0.01 g. Baking loss was calculated according to the method reported by Salehi and Vejdanivahid (2025c).

### 2.6 Volume and Density Determination

Pancake density was determined as an important indicator of internal structure and aeration. The volume of each baked pancake sample was first measured using the canola seed displacement method, which allows accurate determination of irregular‐shaped food products without structural deformation. The mass of each sample was then recorded using a precision digital balance. Density was calculated by dividing the mass of the pancake by its corresponding volume (kg/m^3^).

### 2.7 Puncture Test

The hardness of the carrot pancake surface was determined using a puncture test conducted with a texture analyzer (Santam, STM‐5, Iran). Measurements were carried out using a cylindrical probe (0.5 cm in diameter) at a constant penetration rate of 1 mm/s to a depth of 1 cm. This procedure enabled quantitative evaluation of the force required to deform the pancake surface, serving as an objective indicator of textural firmness.

### 2.8 Moisture Content Determination

The moisture level of the carrot pancake samples was measured using a digital moisture analyzer (DBS60‐3, Kern, Germany). Samples were weighed and subjected to controlled drying conditions, after which the instrument automatically calculated and expressed the moisture content as a percentage based on the mass reduction during the drying process.

### 2.9 Ash Content Determination

Ash content of the carrot pancake samples was determined by weighing 3 g of each sample, initially combusting it over a gas flame, and then incinerating in a muffle furnace (Pars‐Azma‐Co., Iran) at 600 C until constant weight (Samary et al. 2025a).

### 2.10 pH and Acidity Determination

The pH and acidity of the samples were assessed following the protocol described by Vejdanivahid and Salehi (2025). For pH measurement, 10 g of sample was mixed with 100 mL of distilled water and allowed to stand for 20 min for sedimentation. The pH of the supernatant was measured using a calibrated pH meter (Model 827 pH lab, Metrohm, Switzerland) using pH 4 and 7 buffers. For acidity determination, 10 g of powdered sample was placed in a 125 mL Erlenmeyer flask and mixed with 50 mL of 67% ethyl alcohol. After 5 min of stirring, the supernatant was filtered through filter paper. Subsequently, 25 mL of the filtrate was titrated with 0.1 N sodium hydroxide solution (Merck, Germany) using 3 drops of 3% phenolphthalein indicator. The endpoint was indicated by the appearance of a pink color, and the titrant volume was recorded to calculate the sample's acidity using Equation (1):

(1) $$\text{Acidity}\;\left(\%\right)=\frac{2\times \text{V}\times 1.8}{\text{M}}$$

In this equation, V denotes the volume of NaOH solution used in the titration (mL), and M represents the mass of the sample (10 g).

### 2.11 TPC and AC Determination

The TPC of carrot pancakes was assessed following the methods described by Samary et al. (2025a). TPC was determined by extracting 2 g of sample with 80% methanol, reacting the supernatant with Folin–Ciocalteu reagent and sodium carbonate, and measuring absorbance at 725 nm.

AC of the pancakes was assessed using the DPPH assay (Vejdanivahid and Salehi 2025). Extracts were prepared with 80% methanol, mixed with DPPH solution, incubated in the dark at 25°C, and absorbance was measured at 517 nm using a spectrophotometer (XD‐7500, Lovibond, Germany).

### 2.12 Sensorial Evaluation of Pancake

This study was conducted in adherence with the ethical principles outlined in the 1964 Declaration of Helsinki and its subsequent amendments. The research procedures complied with the established ethical standards for investigations involving human subjects. Sensory evaluation of the carrot pancakes was conducted at the Laboratory of New Technologies, Bu‐Ali Sina University, using a panel of 20 participants from diverse age groups. All participants received comprehensive information regarding the study's objectives and methodologies. Prior to their involvement, informed consent was obtained from each participant, ensuring their voluntary and knowledgeable participation. For sensory evaluation, a 9‐point hedonic scale was utilized, with scores ranging from 1 (dislike extremely) to 9 (like extremely). Attributes assessed included appearance, aroma, flavor, texture, and overall acceptability, rated on structured evaluation forms to ensure consistency and reliability.

### 2.13 Statistical Analysis

All experiments were performed using a completely randomized design with three independent replicates for each treatment, where each replicate was prepared and analyzed separately to ensure experimental reliability and reproducibility. The obtained data are presented as mean ± standard deviation. Statistical analysis was conducted using one‐way ANOVA in SPSS (version 21, USA) to evaluate differences among treatments, and significant differences between mean values were determined using Duncan's multiple range test at *p* < 0.05.

## 3 Results and Discussion

### 3.1 Impact of BSG on Batter Color

The effect of BSG concentration on the color indices of carrot pancake batter is presented in Table 1. The results showed that increasing the level of BSG significantly affected all color parameters (*p* < 0.05). The lightness (L*) of the batter increased progressively with the addition of BSG, from 58.18 in the control sample (0.0% BSG) to 66.34 at 0.3% BSG. This increase indicates that BSG incorporation led to a lighter batter appearance, which may be attributed to the high water‐holding capacity of the gum and its ability to modify light reflection by altering the batter matrix structure.

**TABLE 1:** Effect of basil seed gum concentration on the color indices of carrot pancake batter.

Basil seed gum	Lightness	Redness	Yellowness
0.0%	58.18 ± 0.34^c^	27.71 ± 0.12^c^	57.56 ± 0.43^c^
0.1%	61.75 ± 0.64^b^	28.68 ± 0.07^b^	58.31 ± 0.07^c^
0.2%	65.42 ± 0.65^a^	28.85 ± 0.63^b^	64.79 ± 0.63^b^
0.3%	66.34 ± 0.39^a^	30.81 ± 0.09^a^	65.79 ± 0.09^a^

*Note:* Results are expressed as mean values ± standard deviation (*n* = 3). Different letters within each column indicate significant differences among samples (*p* < 0.05).

Similarly, redness (a*) values exhibited a significant upward trend as the BSG concentration increased. The a* value rose from 27.71 in the control sample to 30.81 at 0.3% BSG. This enhancement in redness may be associated with improved dispersion and stabilization of carrot pigments within the batter system in the presence of BSG.

The yellowness (b*) parameter also increased significantly with increasing gum concentration, from 57.56 in the control to 65.79 at the highest BSG level. The simultaneous increase in both a* and b* values suggests that BSG contributed to intensifying the overall color of the carrot pancake batter, potentially by influencing pigment retention and reducing pigment dilution effects during mixing.

### 3.2 Impact of BSG on Color Parameters of Baked Pancakes

The effect of BSG concentration on the color indices of baked carrot pancakes is summarized in Table 2. The results demonstrated that BSG significantly influenced the color attributes of both the surface and core of pancakes (*p* < 0.05), although the magnitude and trend of changes differed between these two regions.

**TABLE 2:** Effect of basil seed gum concentration on the color indices of carrot pancake.

Basil seed gum	Lightness		Redness		Yellowness	
	Surface	Core	Surface	Core	Surface	Core
0.0%	62.84 ± 0.18^a^	59.98 ± 0.68^b^	23.81 ± 0.56^c^	32.40 ± 0.39^b^	60.69 ± 0.55^c^	59.21 ± 0.86^c^
0.1%	62.66 ± 0.36^a^	59.89 ± 0.57^b^	25.98 ± 0.82^b^	32.68 ± 0.16^b^	62.00 ± 1.56^bc^	60.58 ± 0.20^b^
0.2%	59.71 ± 2.28^b^	62.62 ± 0.25^a^	27.66 ± 1.06^ab^	33.58 ± 0.20^a^	63.37 ± 1.15^ab^	62.73 ± 0.76^a^
0.3%	59.34 ± 0.48^b^	63.46 ± 0.55^a^	29.06 ± 0.18^a^	33.83 ± 0.55^a^	65.18 ± 0.27^a^	63.18 ± 0.14^a^

*Note:* Results are expressed as mean values ± standard deviation (*n* = 3). Different letters within each column indicate significant differences among samples (*p* < 0.05).

Regarding lightness (L*), the surface showed a decreasing trend with increasing BSG concentration. The L* value of the surface decreased from 62.84 in the control sample to 59.34 at 0.3% BSG, indicating a darker surface at higher gum levels. This reduction in surface lightness may be attributed to enhanced Maillard reactions and caramelization during baking, possibly promoted by changes in moisture retention and heat transfer induced by BSG. In contrast, the core lightness increased with BSG addition, rising from 59.98 in the control to 63.46 at 0.3% BSG, suggesting a lighter internal structure, which can be associated with improved water distribution and a more uniform core matrix. Hamdani et al. (2021) reported that the incorporation of guar gum and locust bean gum into gluten‐free cookie formulations not only produced lighter‐colored cookies but also improved dough viscosity, viscoelastic properties, texture, and antioxidant activity, demonstrating the beneficial effects of seed gum hydrocolloids on the overall quality of bakery products.

The redness (a*) values of both surface and core increased significantly as the BSG concentration increased. The surface a* value rose from 23.81 (0.0% BSG) to 29.06 at 0.3% BSG, while the core redness increased from 32.40 to 33.83 over the same concentration range. The increase in redness may be related to better retention and stabilization of carrot‐derived pigments as well as intensified non‐enzymatic browning reactions at higher gum concentrations.

Similarly, yellowness (b*) values were significantly affected by BSG incorporation. In the surface, b* values increased from 60.69 in the control to 65.18 at 0.3% BSG, whereas the core exhibited a more pronounced increase, from 59.21 to 63.18. The higher b* values in the core indicate enhanced color saturation, likely resulting from improved pigment dispersion and reduced pigment degradation within the internal structure of the pancake.

Overall, these findings suggest that BSG modifies the color characteristics of baked carrot pancakes in a region‐specific manner. While higher BSG levels tend to darken the surface due to intensified browning reactions, they simultaneously improve core lightness and enhance redness and yellowness in both surface and core. Such changes in color attributes are important quality indicators that can positively influence consumer perception and product acceptability. Ghorbani et al. (2018) investigated the effects of BSG and tragacanth gum on improving the quality and shelf life of fried donuts. The results indicated that the incorporation of each gum at a level of 1% increased moisture content and reduced crumb firmness at both 2 h and 1 week after baking. Moreover, the simultaneous addition of BSG (1%) and tragacanth gum (0.6%) led to an increase in specific volume, a reduction in firmness, an extension of shelf life, higher sensory attribute scores, and improved color indices in the donuts.

### 3.3 Impact of BSG on Physical Characteristics of Baked Pancakes

Table 3 presents the effect of BSG concentration on the physical characteristics of baked carrot pancakes. The results indicate that increasing BSG levels significantly influenced all measured physical parameters, including weight, baking loss, volume, density, and hardness (*p* < 0.05).

**TABLE 3:** Effect of basil seed gum concentration on the physical characteristics of carrot pancake.

Basil seed gum	Weight (g)	Baking loss (%)	Volume (cm^3^)	Density (kg/m^3^)	Hardness (N)
0.0%	22.92 ± 0.06^d^	8.32 ± 0.23^a^	23.02 ± 0.22^d^	995.54 ± 6.87^a^	0.56 ± 0.03^a^
0.1%	23.24 ± 0.04^c^	7.03 ± 0.17^b^	24.29 ± 0.27^c^	957.02 ± 9.04^b^	0.45 ± 0.01^b^
0.2%	23.93 ± 0.06^b^	4.29 ± 0.23^c^	25.44 ± 0.25^b^	940.71 ± 6.85^bc^	0.44 ± 0.04^b^
0.3%	24.63 ± 0.07^a^	1.48 ± 0.29^d^	26.58 ± 0.45^a^	926.87 ± 12.92^c^	0.35 ± 0.01^c^

*Note:* Results are expressed as mean values ± standard deviation (*n* = 3). Different letters within each column indicate significant differences among samples (*p* < 0.05).

BSG exhibits superior stability and enhanced emulsifying and gelling properties compared to many other plant‐derived gums. As a natural, water‐soluble polysaccharide, BSG has attracted considerable attention as a promising functional ingredient due to its excellent emulsification capacity and strong water‐holding ability, making it highly suitable for use in food systems requiring moisture retention and structural stability (Guan et al. 2023). The weight of the pancakes increased slightly but significantly with increasing BSG concentration, from 22.92 g in the control sample to 24.63 g at 0.3% BSG. This rise is likely due to the strong water‐retention ability of BSG, which minimizes water loss during baking and results in a greater final product mass. Kelki Bakhshayesh et al. (2019) investigated the impact of Balangu seed gum on the properties of carrot powder‐enriched sponge cakes. Their results indicated that increasing the gum concentration led to higher post‐baking cake weights, which is consistent with the findings of the present study.

A pronounced reduction in baking loss was observed with increasing BSG levels. Baking loss decreased from 8.32% in the control to 7.03% at 0.1% BSG, followed by a substantial reduction to 4.29% and 1.48% at 0.2% and 0.3% BSG, respectively. This significant decrease demonstrates the ability of BSG to retain water within the pancake matrix during thermal processing, thereby minimizing moisture evaporation.

The volume of pancakes increased significantly as the BSG concentration increased. The volume rose from 23.02 cm^3^ in the control to 26.58 cm^3^ at the highest gum level. This improvement in volume may be associated with enhanced batter viscosity and gas retention capacity, allowing the structure to better entrap air and leavening gases during cooking. Hajmohammadi et al. (2014) evaluated the effect of tragacanth gum incorporation on the quality attributes of sponge cake. The reported results demonstrated that the addition of 0.4% tragacanth gum significantly increased cake volume. Furthermore, during storage, cakes containing tragacanth gum exhibited a softer texture and superior sensory properties compared to the control sample.

Consistent with the increase in volume, the density of pancakes decreased significantly with BSG addition. The density declined from 995.54 kg/m^3^ in the control sample to 926.87 kg/m^3^ at 0.3% BSG. Lower density values indicate a more aerated and porous structure, which is a desirable quality attribute in pancake products. The significant increase in the volume and subsequent decrease in density of the pancakes can be attributed to the hydrocolloid's influence on batter rheology and its ability to stabilize gas cells during baking (Salehi 2020; Salehi and Vejdanivahid 2025a). As a hydrocolloid, BSG can alter the viscosity and viscoelastic properties of the batter, enabling it to trap and retain gases produced during leavening. This enhanced gas cell stabilization prevents premature collapse and promotes a lighter, more aerated final product, ultimately leading to increased volume and lower density. Sanchez‐Pardo et al. (2010) incorporated an oat β‐glucan‐enriched product in combination with dextrin and modified starch into cake formulations to evaluate its effects on quality attributes. Their results indicated an increase in cake volume accompanied by a reduction in density. Furthermore, sensory evaluation revealed that the enriched samples received higher scores compared to the control cakes.

Hardness values showed a marked decrease as BSG concentration increased. The hardness of the control sample was 0.56 N, which decreased to 0.45 N at 0.1% BSG, 0.44 N at 0.2% BSG, and reached the lowest value of 0.35 N at 0.3% BSG. The reduction in hardness can be attributed to improved moisture retention and a more open core structure, resulting in a softer texture. The reduction in hardness observed with increasing BSG concentration is also closely associated with the increased product volume and the corresponding decrease in density, which together contribute to the formation of a more porous and softer pancake structure. The study conducted by Kelki Bakhshayesh et al. (2019) demonstrated that the incorporation of Balangu seed gum reduced both the density and firmness of carrot‐enriched sponge cakes. These findings align with the current research, highlighting the gum's softening effect on cake texture. Sahraiyan et al. (2014) investigated the effects of Balangu Shirazi gum on the quality characteristics of semi‐voluminous gluten‐free bread formulated with sorghum. Their findings indicated that the sample containing 0.5% gum exhibited the highest specific volume and overall acceptability score in sensory evaluation. Moreover, the greatest porosity and the lowest crumb firmness were observed in samples containing 0.25% and 0.5% gum. Effect of hydrocolloid addition on cake prepared with aquafaba as egg substitute reported that the type and concentration of hydrocolloids significantly influenced cake quality, including crumb structure, moisture distribution, and texture. In particular, higher levels of xanthan gum altered the internal cake structure and moisture distribution between the crust and crumb, resulting in a softer cake core. These findings suggest that the functional properties of hydrocolloids are highly dependent on their concentration and their interactions with other ingredients in the formulation, which is consistent with the changes observed in the present study (Crawford et al. 2024).

Overall, the results demonstrate that BSG effectively enhances the physical quality of carrot pancakes by improving moisture retention, increasing volume, reducing density, and producing a softer texture. These improvements are particularly evident at higher BSG concentrations, highlighting the functional potential of BSG as a natural hydrocolloid for the development of bakery products with improved quality attributes.

### 3.4 Impact of BSG on Moisture and Ash Contents of Baked Pancakes

Table 4 presents the moisture and ash contents of carrot powder‐enriched pancakes formulated with different concentrations (0.0% to 0.3%) of BSG. The results demonstrate a significant and concentration‐dependent influence of BSG on both physicochemical parameters.

**TABLE 4:** Moisture and ash contents of carrot pancake containing varying levels of basil seed gum.

Basil seed gum	Moisture (%)	Ash (%)
0.0%	36.49 ± 0.29^d^	1.28 ± 0.03^c^
0.1%	43.45 ± 0.09^c^	1.35 ± 0.02^b^
0.2%	45.76 ± 0.18^b^	1.39 ± 0.01^a^
0.3%	47.58 ± 0.11^a^	1.42 ± 0.01^a^

*Note:* Results are expressed as mean values ± standard deviation (*n* = 3). Different letters within each column indicate significant differences among samples (*p* < 0.05).

The moisture content of the pancakes exhibited a pronounced and statistically significant (*p* < 0.05) increasing trend with the incremental addition of BSG. The control sample (0.0% BSG) had the lowest moisture content at 36.49% ± 0.29%. This value increased sharply to 43.45% ± 0.09% at 0.1% BSG, and continued to rise to 45.76% ± 0.18% and 47.58% ± 0.11% at BSG concentrations of 0.2% and 0.3%, respectively. Notably, the moisture content at 0.3% BSG was approximately 30.4% higher than that of the control. This definitive increase can be attributed to the exceptional water‐holding capacity and hydrocolloid nature of BSG. BSG is composed of hydrophilic polysaccharides that form a three‐dimensional network, physically entrapping and binding free water within the pancake matrix during batter preparation and baking (Naji‐Tabasi and Razavi 2017; Salehi 2020). Consequently, the loss of moisture via evaporation during the baking process is effectively reduced. Higher gum concentrations provide more binding sites and a stronger gel network, leading to greater moisture retention. This result is consistent with findings from studies on other hydrocolloids, such as xanthan or guar gum, in baked goods (Salehi 2020). From a sensory perspective, this retained moisture is likely to contribute positively to the perceived softness, moist mouthfeel, and potentially extend the shelf‐life by delaying staling (Salehi 2020; Salehi and Vejdanivahid 2025a). According to Kelki Bakhshayesh et al. (2019), higher levels of Balangu seed gum resulted in increased moisture content and cake volume in carrot powder‐enriched sponge cakes. These outcomes are in agreement with the present study, demonstrating the positive effect of the gum on the physical characteristics of the cakes.

The ash content, representing the total mineral residue, also showed a significant (*p* < 0.05) increase with rising levels of BSG. The control pancake contained 1.28% ± 0.03% ash. The addition of 0.1% BSG raised the ash content to 1.35% ± 0.02%, followed by further increases to 1.39% ± 0.01% and 1.42% ± 0.01% at 0.2% and 0.3% BSG levels, respectively. While the absolute increase is smaller compared to moisture, the progression is clear and consistent. The increase in ash content is a direct reflection of the mineral composition present in BSG itself. Plant‐based gums often contain inherent minerals such as calcium, potassium, magnesium, and phosphorus (Shahrajabian and Sun 2023). Therefore, as the proportion of BSG in the dry ingredient mix increases, the total mineral contribution from this ingredient increases, leading to a higher measurable ash content after combustion. This finding confirms that BSG not only modifies functional properties but also contributes nutritionally by augmenting the mineral profile of the final product. In a study, the effects of xanthan gum and carboxymethyl cellulose incorporation on the chemical, sensory, and staling properties of cake were investigated. The results of chemical analyses revealed that samples containing these gums exhibited higher moisture and ash contents compared to the control samples without gum addition (Movahhed et al. 2014).

### 3.5 Impact of BSG on pH and Acidity of Baked Pancakes

Table 5 delineates the significant impact of BSG concentration on the pH and acidity of carrot powder‐enriched pancakes. The results reveal a clear and inverse relationship between BSG level and these two critical chemical parameters.

**TABLE 5:** pH and acidity of carrot pancake containing varying levels of basil seed gum.

Basil seed gum	pH	Acidity (%)
0.0%	6.97 ± 0.04^a^	0.20 ± 0.02^d^
0.1%	6.85 ± 0.01^b^	0.28 ± 0.02^c^
0.2%	6.75 ± 0.01^c^	0.36 ± 0.03^b^
0.3%	6.40 ± 0.03^d^	0.46 ± 0.01^a^

*Note:* Results are expressed as mean values ± standard deviation (*n* = 3). Different letters within each column indicate significant differences among samples (*p* < 0.05).

The pH of the pancake samples exhibited a statistically significant (*p* < 0.05) decreasing trend with the addition of BSG. The control sample (0.0% BSG) registered the highest pH of 6.97 ± 0.04, indicative of a nearly neutral product. This value decreased progressively to 6.85 ± 0.01, 6.75 ± 0.01, and 6.40 ± 0.03 for BSG concentrations of 0.1%, 0.2%, and 0.3%, respectively. Notably, the pH at the highest gum concentration (0.3%) was approximately 8.2% lower than that of the control, representing a substantial shift towards a more acidic environment. This significant reduction in pH can be attributed primarily to the intrinsic chemical composition of BSG. BSG is a natural polysaccharide that may contain associated organic acids, uronic acid residues, and other acidic components as part of its complex structure. As the concentration of BSG in the batter increases, the contribution of these acidic moieties becomes more pronounced, thereby lowering the overall pH of the system.

Conversely, the titratable acidity (expressed as a percentage) showed a significant (*p* < 0.05) and concentration‐dependent increase. The control sample had an acidity of 0.20% ± 0.02%, which increased to 0.28% ± 0.02%, 0.36% ± 0.03%, and 0.46% ± 0.01% for 0.1%, 0.2%, and 0.3% BSG, respectively. Remarkably, the acidity at 0.3% BSG was 130% higher (or 2.3 times greater) than that of the control sample. Titratable acidity is a direct measure of the total amount of acid present in a food sample. The sharp increase in acidity aligns perfectly with the observed decrease in pH and provides quantitative evidence for the acidic character contributed by BSG (Shahrajabian and Sun 2023). The linear progression suggests a direct, additive effect where each incremental addition of gum contributes a consistent amount of titratable acid to the batter. This result strongly supports the hypothesis that acidic functional groups within the BSG polysaccharide chain are responsible for the chemical shift.

### 3.6 Impact of BSG on TPC and AC of Baked Pancakes

Food hydrocolloids improve the textural and rheological properties of gluten‐free dough, enhance dough handling and gas retention, and contribute to better overall quality and shelf‐life of baked products. In addition, they may provide functional health benefits, including antioxidant activity, glycemic control, cholesterol reduction, improved satiety, and support for gastrointestinal health through modulation of gut microbiota (Irondi et al. 2023). Table 6 illustrates the positive influence of BSG incorporation on the bioactive profile of carrot powder‐enriched pancakes. The data demonstrate a statistically significant (*p* < 0.05) enhancement in both TPC and AC as the concentration of BSG increases from 0.0% to 0.3%.

**TABLE 6:** Total phenolic content and antioxidant capacity of carrot pancake containing varying levels of basil seed gum.

Basil seed gum	Phenolic content (μg galic acid/g)	Antioxidant capacity (%)
0.0%	983.88 ± 3.80^d^	74.06 ± 0.37^c^
0.1%	992.52 ± 2.31^c^	74.64 ± 0.07^bc^
0.2%	1007.32 ± 3.14^b^	75.03 ± 0.11^b^
0.3%	1019.65 ± 1.51^a^	75.73 ± 0.39^a^

*Note:* Results are expressed as mean values ± standard deviation (*n* = 3). Different letters within each column indicate significant differences among samples (*p* < 0.05).

The TPC of the pancakes showed a consistent and significant upward trend with the addition of BSG. Starting from a baseline of 983.88 ± 3.80 μg GAE/g in the control sample, the TPC increased progressively to 1019.65 ± 1.51 μg GAE/g in the sample containing 0.3% BSG. This represents a total increase of approximately 3.6% over the control. The most pronounced incremental jump was observed between 0.2% and 0.3% BSG. This increase in TPC is directly attributable to the inherent phenolic compounds present in BSG. Basil seeds ( *Ocimum basilicum* L.) are known to contain a variety of phenolic acids and flavonoids which contribute to their antioxidant potential (Shahrajabian and Sun 2023). Therefore, the incorporation of BSG acts as a functional fortification, adding these bioactive phenolics to the food matrix. The synergistic effect between the phenolics from carrot powder (primarily β‐carotene and other carotenoids with antioxidant activity) and those from BSG likely contributes to the observed net increase. The progressive rise suggests that the phenolic compounds in BSG are relatively stable under the baking conditions employed and are effectively transferred to the final product.

Mirroring the trend in TPC, the AC of the pancakes also exhibited a significant (*p* < 0.05) concentration‐dependent improvement with BSG addition. The AC increased from 74.06% ± 0.37% in the control to 75.73% ± 0.39% in the 0.3% BSG sample. This corresponds to a net enhancement of about 2.3% in antioxidant activity at the highest gum level. The elevation in AC is a direct functional consequence of the increased phenolic content (Dindar et al. 2026). Phenolic compounds exert antioxidant activity primarily through their ability to donate hydrogen atoms or electrons, thereby neutralizing free radicals and reactive oxygen species (Pourghasemian et al. 2025). The strong positive correlation observed between TPC and AC is expected and well‐documented in food science literature. The magnitude of increase in AC, while statistically significant, is slightly less pronounced in percentage terms compared to the TPC increase. This could be due to the specific composition of phenolics in BSG, which may have varying antioxidant potencies, or to potential interactions between different antioxidant compounds (carotenoids from carrot and phenolics from BSG) within the complex food matrix, which might not be fully additive in the specific assay used.

In conclusion, the incorporation of BSG at levels up to 0.3% significantly boosts the TPC and AC of carrot powder pancakes. This demonstrates that the functional application of BSG for textural modification simultaneously enhances the nutraceutical profile of the final product.

### 3.7 Impact of BSG on Sensorial Attributes of Baked Pancakes

**FIGURE 2:**
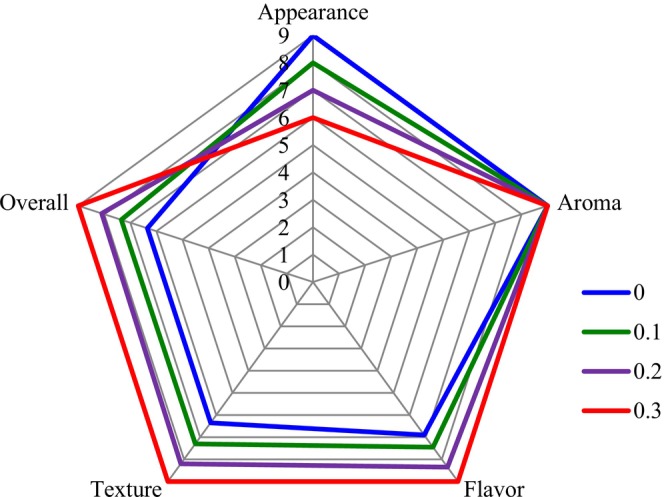
Sensory properties of carrot pancake containing varying levels of basil seed gum (data are shown as mean, *N* = 20).

Figure 2 presents the sensory evaluation results of carrot pancakes formulated with varying concentrations of BSG. The data reveal a complex and insightful trend where the addition of BSG induces a trade‐off between visual appeal and hedonic acceptance of flavor, texture, and overall product, with a clear optimal range emerging. A striking dichotomy is observed between the appearance acceptance and all other sensory parameters. Appearance acceptance declined linearly from a score of 9.0 in the reference sample to 6.0 at the maximum BSG level (0.3%). Conversely, aroma acceptance remained consistently high (9.0) across all formulations, indicating that BSG did not impart any undesirable aromatic notes. More importantly, flavor acceptance, texture acceptance, and overall acceptance all demonstrated a pronounced positive correlation with increasing BSG levels. These scores improved markedly from the control (6.9, 6.4, and 6.4, respectively) to their maximum values at 0.3% BSG. In a study conducted by Hafiz and Sheikholeslami (2020), the effects of Persian gum and BSG at concentrations ranging from 0% to 1%, using a central composite design, were evaluated on the physicochemical properties and quality of loaf bread. The results indicated that increasing gum levels in the bread formulation led to a reduction in water activity, while moisture content and overall sensory quality increased.

The enhancement in texture acceptance can be directly linked to the functional role of BSG. As established in previous analyses, BSG significantly increases moisture retention (Table 4). This moisture likely translates into a perceived softer, moister, and more palatable mouthfeel, overcoming the potential dryness or coreliness that might be present in the control or in carrot‐enriched products. The improved flavor acceptance is multifaceted. First, the enhanced texture positively influences flavor perception, as texture and taste are intrinsically linked. Second, the increased moisture content may improve the release and perception of flavor compounds. Third, the mild acidity contributed by BSG (Table 5) could provide a pleasant balancing note, cutting through sweetness and enhancing the overall flavor profile. The synergy of these factors culminates in the dramatic rise in overall acceptance.

The sensory evaluation conclusively demonstrates that the functional improvements driven by BSG (moisture retention, texture modification, acidification) translate into tangible and highly positive consumer‐relevant benefits. The significant increase in overall acceptance scores, despite a drop in visual appeal, indicates that the improvements in in‐mouth sensory attributes (flavor and texture) are far more critical to overall liking in this product category.

## 4 Conclusion

The present study demonstrated that BSG can be successfully utilized as a natural functional ingredient for improving the quality attributes of carrot powder–enriched pancakes. Incorporation of BSG at low levels (up to 0.3%) significantly enhanced the physicochemical, textural, bioactive, and sensory attributes of the final product. The high water‐holding capacity of BSG played a pivotal role in reducing baking loss, increasing moisture retention, and improving pancake volume, which collectively contributed to a softer texture and lower density. These changes are highly desirable in pancake‐type bakery products, where softness and moistness are critical determinants of consumer acceptance. From a nutritional and functional perspective, the increase in ash content suggests that BSG contributes additional minerals to the formulation. Moreover, the significant enhancement of TPC and AC confirms that BSG acts not only as a technological improver but also as a source of bioactive compounds. The observed positive correlation between TPC and antioxidant activity indicates that the phenolic compounds introduced by BSG remain sufficiently stable during baking and effectively enhance the antioxidant profile of the product. Although the addition of BSG led to a reduction in appearance scores, this drawback was offset by substantial improvements in texture, flavor, and overall acceptability. The sensory findings clearly indicate that in‐mouth attributes exert a greater influence on overall consumer preference than visual characteristics in this product category. In conclusion, BSG represents a promising clean‐label hydrocolloid with dual technological and nutritional functionality. Its application in carrot‐based pancakes and similar bakery products offers a viable strategy for developing value‐added, functional foods with improved quality and enhanced health‐related attributes.

## Author Contributions

**Fakhreddin Salehi:** conceptualization, methodology, software, data curation, supervision, formal analysis, validation, investigation, project administration, writing – original draft, writing – review and editing. **Sepideh Vejdanivahid:** methodology, software, data curation, formal analysis, investigation, writing – original draft.

## Funding

This work was supported by a grant from the Bu‐Ali Sina University, Hamedan, Iran (Grant 40346 to Fakhreddin Salehi).

## Disclosure

Author Approval and Responsibility Statement: All authors have read and approved the final version of the manuscript. Fakhreddin Salehi had full access to all of the data in this study and takes complete responsibility for the integrity of the data and the accuracy of the data analysis.

## Ethics Statement

The authors have nothing to report.

## Conflicts of Interest

The authors declare no conflicts of interest.

## Data Availability

All data generated or analyzed during this research are included in this published manuscript.

## References

[fsn372255-bib-0001] Asgari Verjani, S. , M. Salehifar , and S. Shahriari . 2021. “Assess the Effect of Antioxidant and Antimicrobial Gum Basil Seeds and Essential Oil Oregano in Durability Low‐Fat Chocolate Cake.” Food Research Journal 31, no. 1: 17–31.

[fsn372255-bib-0002] Chen, C. , M. Zhang , W. Liu , and Z. Lin . 2022. “Baking Characteristic Improvement and Starch Retrogradation Inhibition of Chinese Pancakes by Hydrocolloids.” Journal of Food Processing and Preservation 46, no. 5: e16529.

[fsn372255-bib-0003] Crawford, K. , W. Kerr , and C. Tyl . 2024. “Effect of Hydrocolloid Addition on Cake Prepared With Aquafaba as Egg Substitute.” International Journal of Food Science & Technology 59, no. 1: 552–559.

[fsn372255-bib-0004] Dehghani Firoozabadi, A. , M. Hojjateslami , S. Yasin Ardekani , and J. Keramat . 2012. “Effect of Adding Plantago Gum on Staling and Sensory Properties of Sponge Cakes.” In Proceedings of the Second National Conference on Food Science and Technology. Islamic Azad University Ghuchan.

[fsn372255-bib-0005] Dindar, H. , N. Isafi , S. Mohammadi‐Aghdam , and O. Ahmadi . 2026. “Simulating the Green Synthesis Process of Selenium Nanoparticles Using Willow Leaf Extract Under Subcritical Water Conditions and Evaluating Their Properties.” Innovative Food Technologies 13, no. 2: 111–124.

[fsn372255-bib-0006] Ghaemi, P. , S. Arabshahi Delouee , M. Alami , and S. H. Hosseini Ghaboos . 2022. “Effect of Whey Protein Concentrate, Soy Protein Isolate and Basil Seed Gum on Some Physicochemical and Sensory Properties of Rice Flour Based Gluten‐Free Batter and Cake.” Journal of Food Science and Technology (Iran) 19, no. 127: 139–154.

[fsn372255-bib-0007] Ghorbani, M. , Z. Sheikholeslami , and A. Aryanfar . 2018. “The Effect of Basil and Tragacanth Gums on Quality and Shelf Life of Doughnut.” Food Research Journal 28, no. 3: 139–151.

[fsn372255-bib-0008] Guan, L. , Y. Ma , F. Yu , et al. 2023. “The Recent Progress in the Research of Extraction and Functional Applications of Basil Seed Gum.” Heliyon 9, no. 9: e19302. 37662748 10.1016/j.heliyon.2023.e19302 PMC10472252

[fsn372255-bib-0009] Hafiz, M. , and Z. Sheikholeslami . 2020. “Optimization of Loaf Bread Formulation Including Farsi and Basil Gum.” Iranian Food Science and Technology Research Journal 16, no. 4: 395–408.

[fsn372255-bib-0010] Hajmohammadi, A. , J. Keramat , M. Hojjatoleslami , and H. Molavi . 2014. “Evaluation Effect of Tragacanth Gum on Quality Properties of Sponge Cake.” Journal of Food Science and Technology 42, no. 11: 1–7.

[fsn372255-bib-0011] Hamdani, A. M. , I. A. Wani , and N. A. Bhat . 2021. “Pasting, Rheology, Antioxidant and Texture Profile of Gluten Free Cookies With Added Seed Gum Hydrocolloids.” Food Science and Technology International 27, no. 7: 649–659. 33353427 10.1177/1082013220980594

[fsn372255-bib-0012] Irondi, E. A. , Y. T. Imam , E. O. Ajani , and E. O. Alamu . 2023. “Natural and Modified Food Hydrocolloids as Gluten Replacement in Baked Foods: Functional Benefits.” Grain & Oil Science and Technology 6, no. 4: 163–171.

[fsn372255-bib-0013] Jamal Asl, M. , A. Jalilzadeh , and H. Shirdel . 2024. “The Effect of Hot Chocolate Fortification With Carrot Powder on Its Quality Characteristics.” Innovative Food Technologies 11, no. 4: 394–407.

[fsn372255-bib-0014] Kausar, T. , S. Laaraj , A. Hussain , et al. 2024. “Use of Dehydrated Carrot ( *Daucus carota* ) Pomace and Almond ( *Prunus dulcis* ) Powder for Partial Replacement of Wheat Flour in Cake: Effect on Product Quality and Acceptability.” Frontiers in Sustainable Food Systems 8: 1443841.

[fsn372255-bib-0015] Kelki Bakhshayesh, A. , S. H. Hosseini Ghaboos , and G. Asadi . 2019. “Improving the Rheological, Physicochemical, Textural and Sensory Characteristics of Sponge Cake Contained Carrot Powder Using Balangu Seed Gum.” Journal of Food Science and Technology (Iran) 16, no. 88: 61–72.

[fsn372255-bib-0016] Kırbaş, Z. , S. Kumcuoglu , and S. Tavman . 2019. “Effects of Apple, Orange and Carrot Pomace Powders on Gluten‐Free Batter Rheology and Cake Properties.” Journal of Food Science and Technology 56, no. 2: 914–926. 30906049 10.1007/s13197-018-03554-z PMC6400728

[fsn372255-bib-0017] Movahhed, S. , S. Ranjbar , and H. Ahmadi Chenarbon . 2014. “Evaluation of Chemical, Staling and Organoleptic Properties of Free–Gluten Cakes Containing Xanthan and Carboxy Methyl Cellulose Gums.” Iranian Journal of Biosystems Engineering 44, no. 2: 173–178.

[fsn372255-bib-0018] Naji‐Tabasi, S. , and S. M. A. Razavi . 2017. “Functional Properties and Applications of Basil Seed Gum: An Overview.” Food Hydrocolloids 73: 313–325.

[fsn372255-bib-0019] Ozcelik, M. M. , S. Aydin , E. Aydin , and G. Ozkan . 2024. “Preserving Nutrient Content in Red Cabbage Juice Powder via Foam‐Mat Hybrid Microwave Drying: Application in Fortified Functional Pancakes.” Food Science & Nutrition 12, no. 2: 1340–1355. 38370060 10.1002/fsn3.3847 PMC10867499

[fsn372255-bib-0020] Peighambardoust, S. H. , R. A. Homayouni , S. Beikzadeh , J. A. M. Asghari , and M. Beikzadeh . 2016. “Effect of Basil Seed Mucilage on Physical, Sensory and Staling Properties of Sponge Cake.” Iranian Journal of Biosystems Engineering 47, no. 1: 1–9.

[fsn372255-bib-0021] Pourghasemian, P. , A. Pourfarzad , and A. Babakhani . 2025. “Kinetic Modeling of Physicochemical Changes in Protein Bars Fortified With Spirulina and Phycocyanin During Storage.” Innovative Food Technologies 13, no. 1: 23–35.

[fsn372255-bib-0022] Sahraiyan, B. , M. Karimi , M. Habibi Najafi , et al. 2014. “The Effect of Balangu Shirazi (Lallemantiaroyleana) Gum on Quantitative and Qualitative of Surghum Gluten Free Bread.” Iranian Journal of Food Science Technology 11, no. 42: 129–139.

[fsn372255-bib-0023] Salehi, F. 2020. “Effect of Common and New Gums on the Quality, Physical, and Textural Properties of Bakery Products: A Review.” Journal of Texture Studies 51, no. 2: 361–370. 31523824 10.1111/jtxs.12482

[fsn372255-bib-0024] Salehi, F. , and S. Aghajanzadeh . 2020. “Effect of Dried Fruits and Vegetables Powder on Cakes Quality: A Review.” Trends in Food Science & Technology 95: 162–172.

[fsn372255-bib-0025] Salehi, F. , and M. Inanloodoghouz . 2023. “Rheological Properties and Color Indexes of Ultrasonic Treated Aqueous Solutions of Basil, Lallemantia, and Wild Sage Gums.” International Journal of Biological Macromolecules 253: 127828. 37924915 10.1016/j.ijbiomac.2023.127828

[fsn372255-bib-0026] Salehi, F. , and S. Vejdanivahid . 2025a. “Effect of Basil Seed Gum Concentration on the Physical, Textural and Sensory Properties of Quinoa Pancakes.” Innovative Food Technologies 13, no. 1: 13–22.

[fsn372255-bib-0027] Salehi, F. , and S. Vejdanivahid . 2025b. “Effect of Guar Gum on Batter Viscosity and the Physical, Textural, and Sensory Properties of Gluten‐Free Pancakes Based on Quinoa Powder.” Journal of Food and Bioprocess Engineering 8, no. 1: 61–68.

[fsn372255-bib-0028] Salehi, F. , and S. Vejdanivahid . 2025c. “Physicochemical, Textural, and Sensory Effects of Replacing Rice Flour With Quinoa Powder in Gluten‐Free Cake Formulations.” Journal of Food and Bioprocess Engineering 8, no. 1: 69–79.

[fsn372255-bib-0029] Samary, K. , F. Salehi , A. Aliverdi , and A. Daraei Garmakhany . 2025a. “Effect of Magnetized Water and Magnetic Field Treatments on the Physicochemical Properties, Total Phenolic and Antioxidant Capacity of Sprouted Oats Flour.” Innovative Food Technologies 12, no. 3: 273–285.

[fsn372255-bib-0030] Samary, K. , F. Salehi , A. Aliverdi , and A. Daraei Garmakhany . 2025b. “Influence of Magnetic Field Treatment of Oat Seeds During Sprouting on the Physicochemical, Textural, and Sensory Properties of Pancakes Made With Sprouted Oats Flour.” Journal of Food Measurement and Characterization 19: 7980–7993.

[fsn372255-bib-0031] Sanchez‐Pardo, M. , E. Jiménez‐García , and I. González‐García . 2010. “Study About the Addition of Chemically Modified Starches (Cross‐Linked Cornstarches), Dextrins, and Oats Fiber in Baked Pound Cake.” Journal of Biotechnology 150: 316–321.

[fsn372255-bib-0032] Sarmasti, M. , M. S. Mojani‐Qomi , and M. S. Zolfaghari . 2023. “Preparation and Quality Characteristics of Gluten‐Free Sponge Cake Using Alfalfa Seed ( *Medicago sativa* L.) Flour.” Journal of Food and Bioprocess Engineering 6, no. 1: 43–48.

[fsn372255-bib-0033] Shahrajabian, M. H. , and W. Sun . 2023. “Five Important Seeds in Traditional Medicine, and Pharmacological Benefits.” Seeds 2: 290–308.

[fsn372255-bib-0034] Vejdanivahid, S. , and F. Salehi . 2025. “Enhancing the Quality and Nutritional Properties of Gluten‐Free Pancakes Using Sprouted Quinoa Flour Treated With Magnetic Fields, Ultrasound, and Infrared Drying.” Food Science & Nutrition 13, no. 7: e70502. 40688604 10.1002/fsn3.70502 PMC12271974

